# Biomembranes undergo complex, non-axisymmetric deformations governed by Kirchhoff–Love kinematicsand revealed by a three-dimensional computational framework

**DOI:** 10.1098/rspa.2021.0246

**Published:** 2021-11

**Authors:** Debabrata Auddya, Xiaoxuan Zhang, Rahul Gulati, Ritvik Vasan, Krishna Garikipati, Padmini Rangamani, Shiva Rudraraju

**Affiliations:** ^1^ Department of Mechanical Engineering, University of Wisconsin-Madison, Madison, WI 53706, USA; ^2^ Department of Mechanical Engineering, University of Michigan, Ann Arbor, MI 48109, USA; ^3^ Department of Mathematics, University of Michigan, Ann Arbor, MI 48109, USA; ^4^ Michigan Institute for Computational Discovery and Engineering, University of Michigan, Ann Arbor, MI 48109, USA; ^5^ Department of Mechanical and Aerospace Engineering, University of California San Diego, La Jolla, CA 92093, USA

**Keywords:** biomembranes, Kirchhoff-Love, endocytosis, FEM, isogeometric analysis, non-axisymmetric

## Abstract

Biomembranes play a central role in various phenomena like locomotion of cells, cell-cell interactions, packaging and transport of nutrients, transmission of nerve impulses, and in maintaining organelle morphology and functionality. During these processes, the membranes undergo significant morphological changes through deformation, scission, and fusion. Modelling the underlying mechanics of such morphological changes has traditionally relied on reduced order axisymmetric representations of membrane geometry and deformation. Axisymmetric representations, while robust and extensively deployed, suffer from their inability to model-symmetry breaking deformations and structural bifurcations. To address this limitation, a three-dimensional computational mechanics framework for high fidelity modelling of biomembrane deformation is presented. The proposed framework brings together Kirchhoff–Love thin-shell kinematics, Helfrich-energy-based mechanics, and state-of-the-art numerical techniques for modelling deformation of surface geometries. Lipid bilayers are represented as spline-based surface discretizations immersed in a three-dimensional space; this enables modelling of a wide spectrum of membrane geometries, boundary conditions, and deformations that are physically admissible in a three-dimensional space. The mathematical basis of the framework and its numerical machinery are presented, and their utility is demonstrated by modelling three classical, yet non-trivial, membrane deformation problems: formation of tubular shapes and their lateral constriction, Piezo1-induced membrane footprint generation and gating response, and the budding of membranes by protein coats during endocytosis. For each problem, the full three-dimensional membrane deformation is captured, potential symmetry-breaking deformation paths identified, and various case studies of boundary and load conditions are presented. Using the endocytic vesicle budding as a case study, we also present a ‘phase diagram’ for its symmetric and broken-symmetry states.

## 1. Introduction

Membrane curvature is ubiquitous in biology [1]. Indeed, the bending of cell membranes is a central aspect of function for cells and organelles in many cellular processes such as cell migration [2], cell membrane repair [3], membrane trafficking [4] and cytokinesis [5], as well as the maintenance of distinctive membrane shapes within internal organelles like the endoplasmic reticulum [6,7] and the Golgi complex [8]. Some important curved structures include tubules, sheets, vesicles and cisternae [9]. A number of mechanisms have been identified to influence membrane bending, including geometric confinement by protein or lipid components of the membrane (intrinsic factors) [10,11] and peripheral proteins and the cytoskeleton (extrinsic factors) [12,13]. These mechanisms are often coupled and are spatio-temporally regulated by biochemical signalling cascades, leading to the mechanochemical coupling of signalling and membrane deformations. Lipid bilayer models that assume an in-plane fluid-like behaviour and an out-of-plane solid-like behaviour have provided notable insight to investigations of such curvature generation mechanisms. Particularly, the Helfrich–Canham model [14] has furnished mechanistic insight to shape formation of liquid shells during vesiculation [15,16], tubulation [17], viral budding [18], clathrin-mediated endocytosis [19], and membrane neck formation [20,21]. These modelling efforts have been complementary to advances in imaging techniques [22–24], enabling a deeper appreciation of the complexity of membrane deformation.

Despite the wealth of information provided by theoretical membrane mechanics models, an important restriction in several of these studies is the assumption of various degrees of symmetry for the membrane geometry and its deformation. Indeed, the computation of membrane bending phenomena is significantly simplified with the axisymmetric assumption, but as we have shown recently [21], this may come at the cost of generality and precision in identifying the underlying physics, as lower-energy, low-symmetry kinematic modes and even entire mechanisms may be overlooked. With growing interest in curvature-mediated biophysical phenomena and in three-dimensional imaging and reconstruction methods [25,26], there is a need for general purpose computational tools to enable fully three dimensional numerical simulations.

The continuum mechanical treatment of solids considers deformation as a mapping of the geometry (three-dimensional volume, two-dimensional surface or one-dimensional curve) from its reference, undeformed configuration to a deformed current configuration under the influence of internal or external loads, of which the latter also may appear as boundary conditions. In limited cases, the geometry, loads and boundary conditions result in a mathematical problem of deformation of a k-manifold immersed in an n-dimensional space ($R^{n}$). A 3-manifold is a volume, 2-manifold is a surface and 1-manifold is a curve. For k=n, modeling solid deformation is relatively straightforward and can be accomplished in the framework of Euclidean geometry using a rectilinear coordinatebasis.

However, deformation of shell-like surface geometries, as is the case with biological membranes, involves tracking the underlying kinematics and evolution of geometric configurations of a 2-manifold embedded in a three-dimensional space [27]. Such a geometric embedding demands a non-Euclidean framework with a curvilinear coordinate basis. While the mathematical treatment of such a framework is well-developed (beginning with the celebrated work on differential geometry by Riemann in the nineteenth century [28]), its application to three-dimensional modelling of biomembranes, which entails solving nonlinear partial differential equations in a curvilinear coordinate basis, is relatively recent. Beginning with finite element models of Mindlin–Reissner plates [29–32] and Kirchhoff–Love shells [29,33–35], initial efforts focused on developing numerical models in a rectilinear coordinate basis with approximated geometries and kinematics. However, the advent of spline-based geometric representations of surfaces and the more recent development of Isogeometric Analysis (IGA) techniques [36] allow for an exact representation of surface geometries and the use of a curvilinear coordinate basis. Such treatments are now gaining traction in modelling structural applications [37–40] and also in the context of biological materials [41–44]. We build upon these developments, especially from Sauer *et al.* [42], by adopting spline-based representations of surface geometries, treatments of membrane kinematics using a curvilinear basis, and the framework of IGA to develop a comprehensive computational modelling framework for studying complex deformations in biological membranes.

In this work, we present a three-dimensional, Helfrich-energy-based, Kirchhoff–Love thin-shell computational framework for modelling the deformation of biological membranes in the regime of fully nonlinear kinematics and precise geometric representations. With this treatment, we are able to model membrane deformations, resolve geometric bifurcations, and explore post-bifurcation responses. The main ingredients of this framework are the governing equations of Helfrich-energy-based membrane mechanics [27,42,44,45] and the numerical framework of IGA for solving the underlying partial differential equations. IGA methods form a numerical framework for finding approximate solutions to general partial differential equations [36], are a generalization of the classical finite-element method [46–48], and possess good numerical approximation and stability properties [49]. Crucially for accurate modelling of membrane biophysics, since IGA uses spline basis functions to represent the geometry and its deformation, it admits the continuity of slopes that is a characteristic of membranes in all states except for those of actual scission. As a result, we can now investigate simulations of membrane deformation under conditions that are notably more general (having fewer restrictive kinematic assumptions) than those considered previously in the literature [20,50–54]. The computational framework is implemented as an open-source software library and provided as a resource to the biophysics community [55].

**Figure 1.**
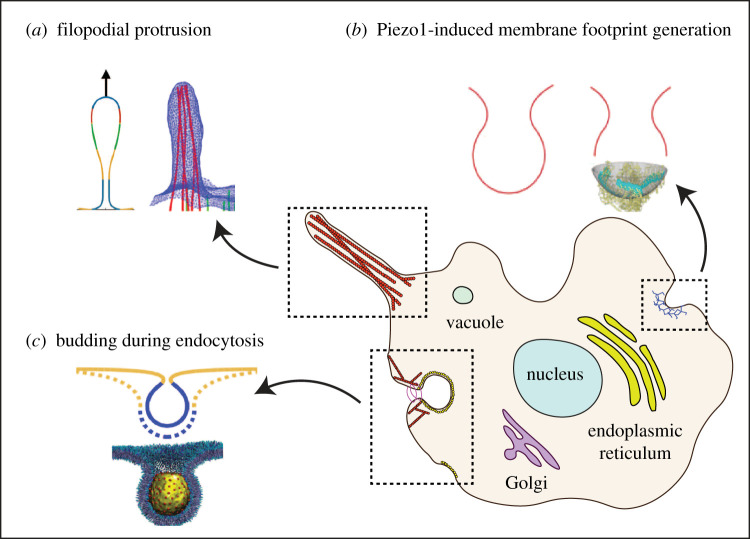
Schematic of the various membrane biophysical phenomena modelled in this work to demonstrate the computational framework: (*a*) membrane tube pulling during filopodial protrusion, (*b*) dome formation and membrane footprint generation due to Piezo1 interaction, and (*c*) spontaneous curvature-driven bud formation during endocytosis. Shown in insets are the schematic of the membrane deformation induced by the underlying protein complexes and its line diagram representation. (Online version in colour.)

To demonstrate the scope of the computational framework, we simulate three classical and non-trivial membrane deformation phenomena (figure 1): (a) formation of tubular shapes and their lateral constriction, (b) Piezo1-induced membrane footprint generation and gating response and (c) the budding of membranes due to the spontaneous curvature of the protein coats during endocytosis. For each case, three-dimensional membrane deformation is tracked, symmetry-breaking deformation pathways identified, and a few case studies of boundary conditions and loading are presented to exhibit the fidelity and modelling potential of the proposed methodology. We also present a phase diagram of symmetric and broken-symmetry states of membrane budding during endocytosis.

In the following sections, we present an outline of the mathematical framework and the model development, followed by a presentation of the three boundary value problems considered, their modelling results and biophysical implications. Finally, a discussion of the framework, its utility and planned future developments is presented.

## 2. Methods

The mathematical framework consists of surface geometry parametrization, Kirchhoff–Love membrane kinematics, Helfrich-energy-based mechanics of lipid bilayers and surface partial differential equations governing mechanical deformation. Key ingredients of this framework are described below, while the more detailed mathematical derivations are provided in the electronic supplementary material. Using the IGA apparatus, the mathematical treatment is then cast into a numerical formulation that allows for solving the governing equations to obtain the spatial evolution of membrane deformation. These aspects of the framework are discussed under the computational implementation subsection.

### (a) Mathematical framework

The mathematical treatment introduced here follows from Sauer *et al.* [42]. Only the important results are summarized in this section, and the detailed derivations are presented in the electronic supplementary material.

#### (i) Surface parametrization and kinematics

**Figure 2.**
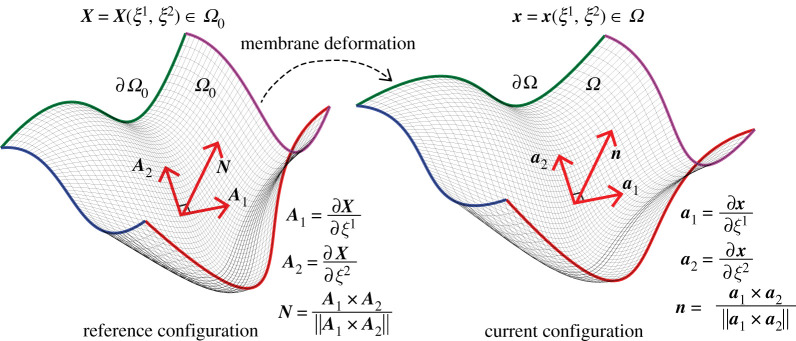
Surface parametrization of a biomembrane in the reference undeformed configuration ($\Omega_{0}$) and current deformed configuration (Ω). The two-dimensional surface, $\Omega_{0}$, is bounded by the curves $\partial \Omega_{0}$ (highlighted with colour), and embedded in a three-dimensional volume. Here, X is the position vector of a point on the surface parametrized in terms of the surface coordinates ($\xi^{1}$,$\xi^{2}$) which are associated with a flat two-dimensional domain that is then mapped to $\Omega_{0}$ as $X=X(\xi^{1},\xi^{2})$. The local tangent vectors to the surface at X are $A_{1}$ and $A_{2}$, and N is the corresponding surface normal. The position dependent triads $\left\{A_{1},A_{2},N\right\}$ and {$a_{1}$,$a_{2}$,n} form the local curvilinear coordinate basis for the reference undeformed configuration and current deformed configuration, respectively. (Online version in colour.)

Consider a lipid bilayer represented as a surface (2-manifold) embedded in a three-dimensional volume, as shown in figure 2. Let the reference (undeformed) configuration and the current (deformed) configuration of the surface geometry be denoted by $\Omega_{0}$ and Ω, respectively. The configurations $\Omega_{0}$ and Ω are parametrized by the coordinates $\xi^{1}$ and $\xi^{2}$ that map a flat two-dimensional domain to the surface coordinates X and x:

2.1 $$X=X(\xi^{1},\xi^{2})\;\forall \;X\in \Omega_{0}\;\text{and}\;x=x(\xi^{1},\xi^{2})\;\forall \;x\in \Omega .$$

The (covariant) tangent vectors in the reference and current configuration are given by

2.2 $$A_{I}=\frac{\partial X}{\partial \xi^{I}}=X{,}_{I}\;\text{and}\;a_{i}=\frac{\partial x}{\partial \xi^{i}}=x{,}_{i}.$$

In the expressions that follow, except when indicated otherwise, uppercase letters are associated with the reference configuration and lowercase letters are associated with the current configuration.

Using the tangent vectors, we define the surface normals as follows:

2.3 $$N=\frac{A_{1}\times A_{2}}{∥A_{1}\times A_{2}∥}\;\text{and}\;n=\frac{a_{1}\times a_{2}}{∥a_{1}\times a_{2}∥}.$$

From the triad consisting of the tangent vectors and the normal that form the local curvilinear coordinate basis, we can obtain expressions for the metric tensor,

2.4 $$A_{IJ}=A_{I}\cdot A_{J}\;\text{and}\;a_{ij}=a_{i}\cdot a_{j}.$$

The second-order derivatives of the surface coordinates X and x are given by

2.5 $$A_{I,J}=\frac{\partial A_{I}}{\partial \xi_{J}}\;\text{and}\;a_{i,j}=\frac{\partial a_{i}}{\partial \xi_{j}},$$

and from them we obtain the components of the curvature tensor,

2.6 $$B_{IJ}=A_{I,J}\cdot N\;\text{and}\;b_{ij}=a_{i,j}\cdot n.$$

We are now able to define the primary kinematic metrics of interest: the mean and Gaussian curvature. The mean curvature and Gaussian curvature are frame invariant measures of a surface geometry, and hence are natural choices for representing the kinematics of the surface as it deforms. Using the components of the curvature tensor, we can obtain expressions for the mean curvature,

2.7 $$H=\frac{1}{2}B^{IJ}A_{IJ}\;\text{on}\;\Omega_{0},\;h=\frac{1}{2}b^{ij}a_{ij}\;\text{on}\;\Omega ,$$

and the Gaussian curvature,

2.8 $$K=\frac{|B|}{|A|}\;\text{on}\;\Omega_{0},\;\kappa =\frac{|b|}{|a|}\;\text{on}\;\Omega ,\;|\cdot |=det(\cdot ).$$

#### (ii) Biophysics of membrane deformation

With a focus on representing the correct deformation, a biomembrane is often modelled as a thin elastic shell governed by the classical Helfrich formulation [14,56,57] of membrane bending energy. In this treatment, the primary kinematic variables are the curvatures capturing the bending of the membrane, and the elastic energy density of the membrane is given by

2.9 $$w=k_{B}{\left(h-h_{0}\right)}^{2}+k_{G}\kappa ,$$

where $k_{B}$ and $k_{G}$ are the bending modulus and the Gaussian curvature modulus of the membrane, and $h_{0}$ represents the instantaneous curvature induced in the membrane.

Furthermore, we assume that the membrane is area preserving (i.e the membrane area is constant) [58]—a constraint that is implemented using a Lagrange multiplier field. Enforcing the area-preserving condition results in the following field expression for the elastic energy density:

2.10 $$w=k_{B}{\left(h-h_{0}\right)}^{2}+k_{G}\kappa +\lambda (J-1),$$

where λ is the point value of the Lagrange multiplier field, and J is the surface Jacobian field (ratio of an infinitesimal area element in the current configuration to the area of its pre-image in the reference configuration). Here, the Lagrange multiplier field represents the membrane tension [45,59] that enforces the area-preserving property of biomembranes and thus influences the minimum energy configuration. The Lagrange multiplier field is position dependent, is obtained as part of the solution process, and thus permits non-homogeneous membrane tensions that are needed to ensure that the membrane is area preserving under general deformation conditions. In this model, we neglect in-plane fluidity of the membrane [60,61] and friction in the bilayer [62–64], as we are interested in determining the elastic equilibrium states under quasi-static conditions and not the underlying membrane relaxation or rate processes. The augmented Helfrich Hamiltonian whose extremum is sought over the membrane surface, including the Lagrange multiplier field λ is given as

2.11 $$E=\int_{\Omega }(k_{B}{\left(h-h_{0}\right)}^{2}+k_{G}\kappa +\lambda (J-1))\;\text{d}a,$$

where Ω is the domain of integration over the membrane surface.

#### (iii) Governing equations

The governing equation for quasi-static mechanical equilibrium in three-dimensional simulations is obtained as the Euler–Lagrange condition of the Helfrich energy functional following standard variational arguments, and is given by

2.12 $$\int_{\Omega }\frac{1}{2}\delta a_{ij}\sigma^{ij}\;\text{d}a+\int_{\Omega }\delta b_{ij}M^{ij}\;\text{d}a-\int_{\Omega }\delta x\cdot p\;\text{d}a-\int_{\partial \Omega }\delta x\cdot t\;\text{d}s=0,$$

where ∂Ω is the membrane boundary on which surface tractions and displacement boundary conditions can be applied, as shown in figure 2. Furthermore, $\delta a_{ij}$ and $\delta b_{ij}$ are variations of the components of the metric tensor and the curvature tensor, respectively,

Here, $\sigma^{ij}$ are the components of the stress tensor, $M^{ij}$ are components of the moment tensor (in the current configuration), p is a body force, which can be used to apply surface pressure for constriction (in the case of the tube constriction boundary value problem), and t is the surface traction.

For a hyperelastic material model, we can express the stress and moment components in terms of the strain energy density as [44]

2.13 $$\sigma^{ij}=\frac{2}{J}\frac{\partial w}{\partial a_{ij}}$$

and

2.14 $$M^{ij}=\frac{1}{J}\frac{\partial w}{\partial b_{ij}}.$$

For the Helfrich type strain energy density, these take the form

2.15 $$\sigma^{ij}=(k_{B}{\left(h-h_{0}\right)}^{2}-k_{G}\kappa )a^{ij}-2k_{B}(h-h_{0})b^{ij}$$

and

2.16 $$M^{ij}=(k_{B}(h-h_{0})+2k_{G}h)a^{ij}-k_{G}b^{ij}.$$

Here, it is important to note that the Helfrich elastic model inherently lacks resistance to shear deformation modes in three dimensions. This lack of shear stiffness correctly represents the fluidity of the biomembranes, but induces numerical instabilities while solving boundary value problems involving three-dimensional membrane deformation. We eliminate these numerical instabilities by adding shear stabilization terms to the material model but ensure that these terms are of smaller magnitude than the bending energy terms in the Helfrich energy [42]. We perform convergence studies with respect to both the underlying mesh (ensuring mesh-objectivity) and the dependence on the shear stabilization terms. The results reported in this manuscript are for sufficiently refined meshes. The elastic modulus corresponding to the shear stabilization is small compared to the bending modulus and is chosen to have minimal effect on the overall stiffness or the deformation energy of the membrane.

### (b) Computational implementation

In this framework, we solve the governing equation given by equation (2.12) using the methodology of Isogeometric Analysis (IGA) [36]. As stated in the introduction, IGA is a mesh-based numerical discretization scheme for finding approximate solutions to general partial differential equations [36], and is a generalization of the classical finite-element method [46–48]. Numerical discretization of the problem geometry in IGA is accomplished by using a spline-based $C^{1}$-continuous basis. In the context of biomembranes, this ensures accurate representation of both the reference and deformed geometries without the spurious slope discontinuities observed in more traditional finite-element schemes and other grid-based numerical schemes. We developed a first of its kind in-house, parallel, C++ programming language-based open-source library for membrane mechanics in three dimensions. The important components of this modular library are the implementation of membrane kinematics without any axisymmetric restrictions, Helfrich material model, weak form of the governing equations of membrane mechanics, and the setup of the global boundary value problem with biomembrane-specific boundary conditions. This library sits on top of the PetIGA [65] open-source library that provides the spline (NURBS) discretization capability and the PETSc [66] open-source library that provides a suite of data structures and routines for the scalable (parallel) solution of partial differential equations. The computational framework is implemented as an open-source software library and is provided as a resource to the biophysics community through a GitHub repository [55].

## 3. Results

We demonstrate the simulation framework using three classical membrane deformation problems: formation of tubular shapes and their lateral constriction, Piezo1-induced membrane footprint generation and gating response, and the budding of membranes by protein coats during endocytosis. Through these examples, we also demonstrate the emergence of increasingly complex membrane deformations that are beyond the scope of traditional axisymmetric formulations. These problems are described in detail below.

### (a) Formation of tubular shapes and their lateral constriction

Many cell organelles and cytoplasmic projections are shaped as vesicles, tubes or elongated membrane structures. Some examples of such shapes are the filopodia protrusions, inner mitochondrial region, endoplasmic reticulum, the Golgi complex, etc. (figure 1). These tubular structures play an important role in the locomotion of cells, production and folding of proteins, and in the formation of vesicles for transporting proteins and lipids among others. A typical mechanism for producing these tubular shapes involves motor proteins that attach to the cell membrane and pull it along the filaments of the cytoskeleton [67,68]. Further, as is the case with the fission of endocytic vesicles, the tubular or vesicular structures also undergo constriction by scission proteins like dynamin [69–71]. This constriction mediates a membrane pinch-off mechanism that leads to the formation of vesicles. From a biophysical standpoint, it is important to gain a quantitative understanding of the interaction between the proteins and the membranes by determining the deformation mechanisms, forces exerted by proteins, and kinematic constraints.

A classic benchmark problem in the understanding of elongated biomembrane structures is the analytical model of the formation and interaction of membrane tubes proposed by Derényi *et al*. [17]. Some key results of this model are the prediction of the magnitude of protein-membrane interaction forces and tubule radius, and their dependence on the membrane bending modulus ($\kappa_{B}$) and surface tension (γ). The protein pulling force, $t_{y}$, and the tubule radius, r, are related to the bending modulus and surface tension of the membrane as follows: $t_{y}\propto \sqrt{\kappa_{B}\;\gamma }$ and $r\propto \sqrt{\kappa_{B}/\gamma }$. In addition to these analytical estimates, numerical solutions to the problem of membrane tube pulling, albeit with axisymmetric constraints on deformation, are available in the literature [72,73] and in our earlier work [21]. We take advantage of the analytical estimates proposed by Derényi *et al.*, the numerical solutions available from axisymmetric models [21], and validate the computational framework proposed in this work by comparing the load–displacement response of membrane tube pulling from these three approaches.

**Figure 3.**
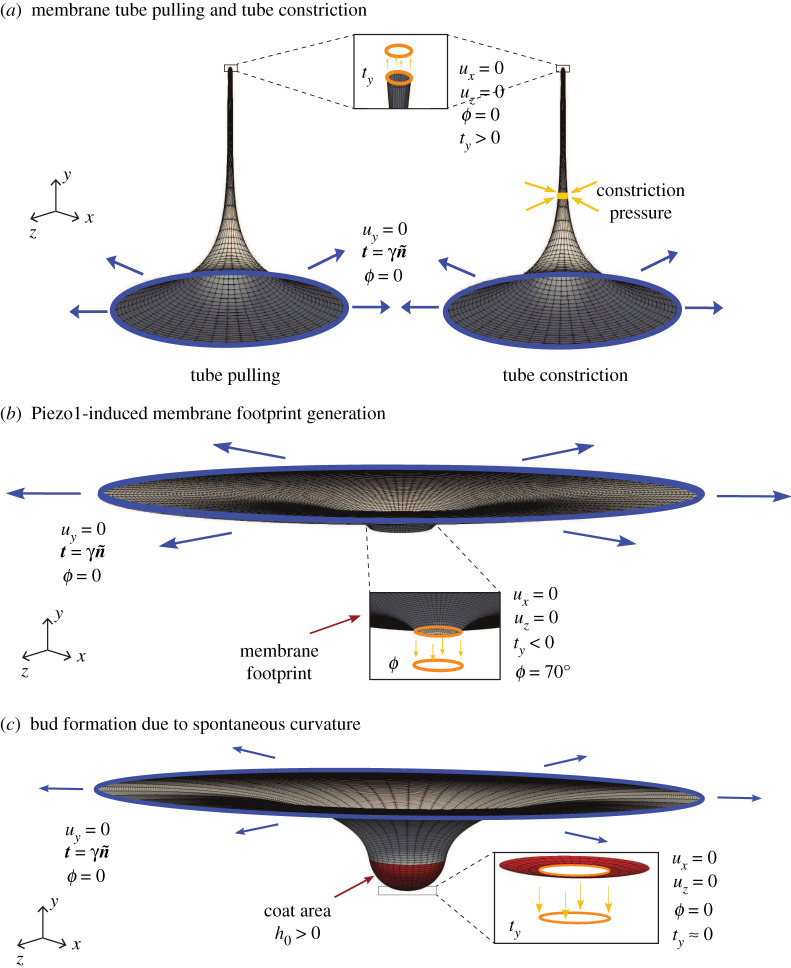
Schematic of the various membrane boundary value problems considered in this work. Shown are the geometry and boundary conditions for (*a*) formation of tubular shapes and their lateral constriction due to the application of axisymmetric constriction pressure, (*b*) Piezo1-induced membrane footprint generation, and (*c*) the budding of membranes due to the spontaneous curvature of the protein coats during endocytosis. Here, $u_{x}$, $u_{y}$ and $u_{z}$ are the displacement components, t is the surface traction and ${\text{t}}_{\text{y}}$ its component along the y-axis, $\tilde{n}$ is the normal to the boundary curve, ϕ is the boundary slope, γ is the surface tension applied on the membrane boundary, and $h_{0}$ is the instantaneous curvature. Blue and orange colours identify the outer and inner rims, respectively. (Online version in colour.)

**Figure 4.**
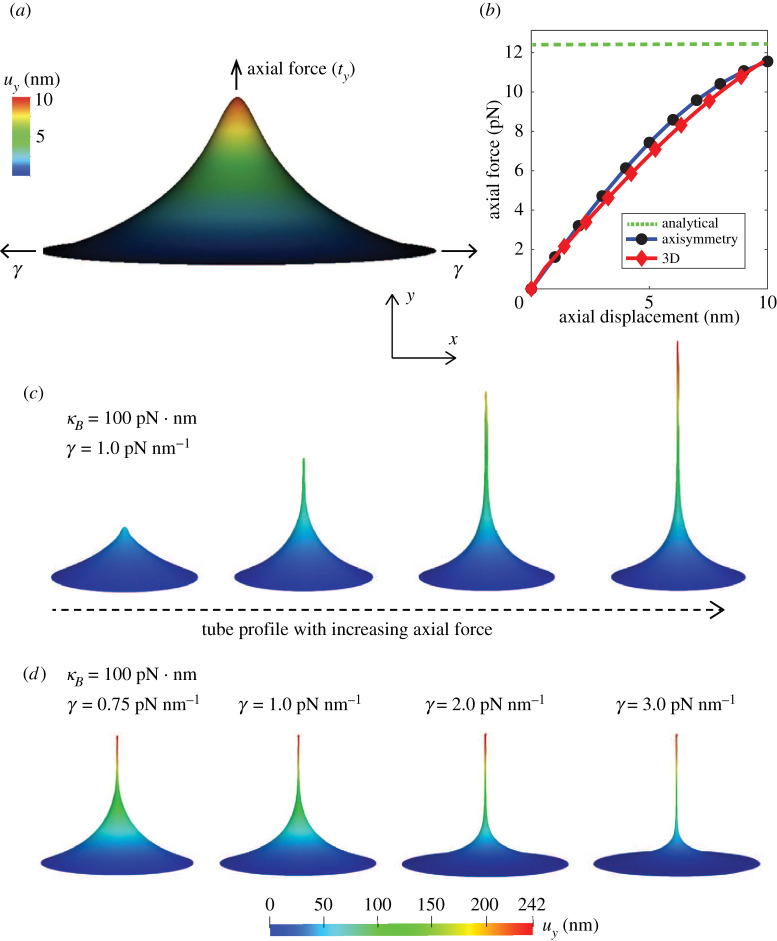
Deformation profile and force–displacement response of a membrane during tube pulling. Shown are the (*a*) deformation profile with the application of axial force ($t_{y}$) on a membrane with a bending modulus ($\kappa_{B}$) of 20 pN · nm under a surface tension (γ) of $0.1\;{\mathrm{pN}\;\mathrm{nm}}^{-1}$, (*b*) comparison of the three-dimensional force–displacement response with the axisymmetric solution and the equilibrium tube pulling force predicted by the analytical model, (*c*) progression of tube pulling with increasing axial force and (*d*) dependence of the deformation profile and tube radius on the surface tension of the membrane. (Online version in colour.)

The boundary value problem solved, along with the spatial discretization (mesh), boundary conditions on the displacement (u) and the membrane boundary slope (ϕ) are shown in figure 3*a*. The simulation results are shown in figure 4: figure 4*a* is the deformation profile obtained during tube pulling, and in figure 4*b* is the load–displacement response of the three-dimensional framework compared to the asymmetric result and the equilibrium value of tube pulling force predicted by the analytical model. We note that the analytical model only predicts the final equilibrium value of the tube pulling force, and hence only a single value of the force from the analytical model is plotted. As can be seen from figure 4*b*, the three-dimensional model very closely tracks the axisymmetric solution and asymptotically approaches the equilibrium value of force from the analytical solution. Furthermore, we show the evolution of the deformation profile with increasing tube pulling force in figure 4*c*, and the dependence of the deformation profile and tubule radius on the applied surface tension in figure 4*d*. Here, we note that the small deviation of the three-dimensional model results from the axisymmetric solution in figure 4*b* is due to the fact that the three-dimensional model boundary value problem is less constrained along the outer rim than the axisymmetric boundary value problem. For the three-dimensional problem, we enforce $u_{y}=0$ along the outer rim, whereas the axisymmetric problem also enforces complete radial symmetry of the $u_{x}$ and $u_{z}$ displacements in addition to enforcing $u_{y}=0$ (see figure 3*a*). This makes the axisymmetric problem more stiff to the applied load.

**Figure 5.**
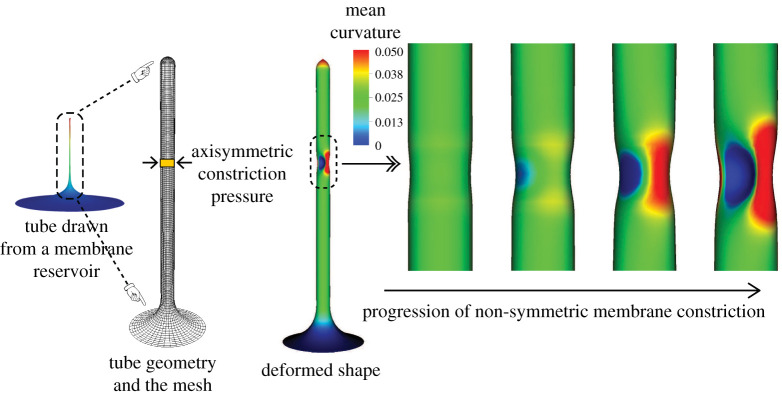
Progression of membrane tube constriction with increasing constriction pressure leading to non-symmetric pinching profiles of deformation. Shown are the tube geometry and the computational mesh, and the progression of the non-symmetric membrane constriction due to the constriction pressure. (Online version in colour.)

We further consider the effect of lateral constriction pressure on the tubular geometry and demonstrate the non-axisymmetric pinching deformation profile that is predicted by the computational framework. For this boundary value problem, we consider a tubular geometry (shown in figure 1*a*, under tube constriction) and apply an axisymmetric constriction pressure that would be applied by a spiral collar protein like dynamin [57,74,75]. As can be expected, an axisymmetric model would predict an axisymmetric pinching profile in the vicinity of the constriction pressure [21]. However, the fully three-dimensional model considered in this computational framework is not limited to axisymmetric solutions, and is thus able to predict non-axisymmetric states when they are the energy minimizing solutions to the governing equations of membrane deformation. The progression of the non-axisymmetric solution with increasing constriction pressure is shown in figure 5. This shape of the membrane has significant implication on the force and energy barrier of protein induced pinching of membranes, as has been studied in detail in our recent work demonstrating how non-axisymmetric buckling lowers the energy barrier associated with membrane neck constriction [21]. In that study, we used an earlier version of the computational framework proposed here to study the influence of location, symmetry constraints and helical forces on membrane neck constriction in a lipid bilayer. Simulations from our model demonstrated that the energy barriers associated with constriction of a membrane neck are location dependent, and if symmetry restrictions are relaxed, the energy barrier for constriction is dramatically lowered and the membrane buckles at lower values of the constriction pressure. These studies helped establish that even though there exist different molecular mechanisms of neck formation in cells, the mechanics of constriction of a cylindrical membrane tubule naturally leads to a loss of symmetry that can lower the energy barrier to constriction. This loss of symmetry may be a common mechanism for different scission processes and clearly demonstrates the need for a fully three-dimensional computational framework to give predictive insights into membrane deformation.

### (b) Piezo1-induced membrane footprint and gating response

We next investigate how mechanosensitive channels can deform the membrane. Mechanosensitive ion channels on the cell membrane play an important role in the mechanosensory transduction processes of the cell. These ion channels are sensitive to the forces acting on the cell membrane and respond to these forces by undergoing conformational changes. These changes result in the opening and closing of pores in the cell membrane and thereby regulate the flow of ions and solutes entering and egressing the cell. Examples of such mechanosensitive ion channels include Piezo1, MscL and TREK-2 [76]. In the case of Piezo1, a gated ion channel made up of three protein subunits that induce a dome-shaped structure on the cell membrane, the gating mechanism is triggered by the membrane surface tension. The membrane deformation induced by the surface tension acts as a mechanical signal that activates the protein subunits and causes them to undergo a conformational change that results in pore opening and transport of ions and solutes [77–79].

While the exact mechanism of mechanosensory transduction effected by the Piezo1 ion channel is still an open question, the extent of the deformed shape induced by the Piezo1 dome (referred to as the membrane footprint) is understood to significantly influence the sensitivity of the gating response of the channel [80]. As observed by Haselwandter & MacKinnon [80], an extended membrane footprint amplifies the sensitivity of Piezo1 subunits to respond to changes in the membrane surface tension. At the same time, increasing membrane tension significantly reduces the membrane footprint and thereby renders the Piezo1 subunits less sensitive to detect membrane mechanical signals.

**Figure 6.**
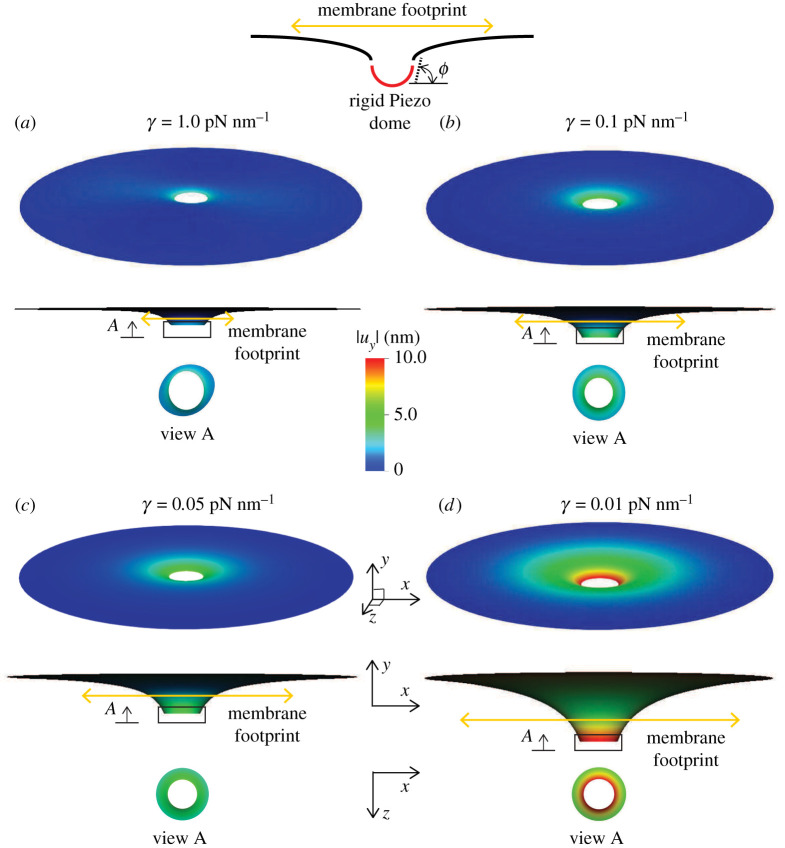
Effect of surface tension on the membrane footprint area induced by a Piezo1 dome. Plotted are the three-dimensional displacement profile, and its projection on the x--y and z--x planes. The bending modulus ($\kappa_{B}$) of the membrane is taken to be 30 pN⋅nm, and a rigid Piezo dome effect is simulated by rotating the membrane (slope boundary condition) at the inner rim of the annular geometry to a value of $\phi =70{\;}^{\circ }$. To clearly visualize the increasing membrane footprint with decreasing surface tension, we scale the y component of the displacement ($u_{y}$) by a factor of three in the x--y oriented plots. (*a*) $\gamma =1.0\;{\mathrm{pN}\;\mathrm{nm}}^{-1}$, (*b*) $\gamma =0.1\;{\mathrm{pN}\;\mathrm{nm}}^{-1}$, (*c*) $\gamma =0.05\;{\mathrm{pN}\;\mathrm{nm}}^{-1}$ and (*d*) $\gamma =0.01\;{\mathrm{pN}\;\mathrm{nm}}^{-1}$. (Online version in colour.)

In this analysis, we model the effect of surface tension on the area of the membrane footprint induced by the Piezo1 dome. Our modelling goal for this problem is to demonstrate the effect of membrane tension on: (1) the membrane footprint, and (2) the out-of-plane membrane displacement that can be interpreted as a kinematic trigger to activate the gating mechanism in the protein subunits of Piezo1. The schematic for this boundary value problem is shown in figure 3*b*, and the simulation results demonstrating the effect of surface tension on the membrane footprint are presented in figure 6. The plots show the three-dimensional displacement profiles and their two-dimensional projections under the boundary conditions enforced by the Piezo1 dome. A Piezo dome effect on the membrane is modelled by rotating the membrane (slope boundary condition) at the inner rim of the annular geometry to a value of $\phi =70{\;}^{\circ }$ that is chosen so as to simulate the effect of a nearly hemispherical dome (which would correspond to $\phi =90{\;}^{\circ }$). This slope boundary condition assumes that the Piezo1 protein complex is a rigid dome that enforces a rotation on the surrounding membrane to ensure slope continuity between the hemispherical dome and the connected membrane. As can be seen from the figure 6*a*–*d*, decreasing the surface tension increases the membrane footprint. Especially, in the limit of very low surface tension ($\gamma =0.01\;{\text{pN nm}}^{-1}$), we see a significantly enhanced membrane footprint. The change in the out-of-plane displacement of the membrane, ($u_{y}$), shows a similar dependence on the surface tension. Since the out-of-plane displacement can be interpreted as a kinematic trigger to activate the gating mechanism in the protein subunits of Piezo1, this implies that at lower surface tension values, a higher value of $u_{y}$ is attained, thus delivering an amplified kinematic trigger, and therefore greater sensitivity of the Piezo1 dome to changes in surface tension. These results are consistent with the observations by Haselwandter & MacKinnon [80] that use the classical reduced order Monge and arc-length axisymmetric parametrization methods to model the Piezo1-induced membrane deformation. Note that the deformation profile at the inner rim is, in general, non-axisymmetric, an effect that increases with membrane tension, γ. This illustrates the power of the three-dimensional computational framework, which while it encompasses axisymmetric deformation, also admits non-axisymmetric modes. With access to the larger space, deformation profiles that are attainable at lower energies are indeed attained since the elasticity problem results in a (local-) minimum energy configuration. Thus, while the three-dimensional model reproduces the trends predicted by the reduced order models, its true power is in identifying more complex deformation patterns that are not accessible to the reduced order axisymmetric models.

### (c) Budding of membranes by protein coats during endocytosis

Budding of membranes by protein coats is a critical process in clathrin-mediated endocytosis (CME) that transports substance from the extracellular matrix to the cell interior. Several key features, including protein-induced spontaneous curvature, membrane properties, membrane tension and force from actin polymerization, have been identified to govern the bud formation in CME [19,81,82]. The ability to simulate the morphological progression of bud formation in the three-dimensional setting under different combinations of these identified features is crucial for understanding the mechanical progression of CME. To further demonstrate the predictive capability of our simulation framework, we investigate the relationships between coat area, coat curvature and degrees of symmetry during the budding of a vesicle as part of the endocytosis phenomena. The schematic of the simulation set-up is given in figure 3*c*. We simulated bud formation under two different conditions in our framework, similar to the setup explored in Hassinger *et al.* [19]. In the first case (i), the coated region has a fixed spontaneous curvature $h_{0}=0.02\;{\text{nm}}^{-1}$ with progressively increasing area of the coat. In the second case (ii), the coated region has a fixed radius of 80 nm with progressively increasing spontaneous curvature. In both cases, the uncoated membrane has a radius of 400 nm and has a surface tension with $\gamma =0.002\;{\text{pN nm}}^{-1}$. The bending modulus ($k_{B}$) for all cases is taken to be 320 pN⋅nm. The slope boundary condition with ϕ=0 is enforced at both the inner and outer rims of the membrane through the penalty method to ensure the continuous differentiability with the flat membrane reservoir. As illustrated in figure 3*c*, Dirichlet boundary conditions are enforced to eliminate rigid body motions. The hyperbolic tangent function proposed in Hassinger *et al.* and Rangamani *et al.* [19,83] (see electronic supplementary material for illustration of this function) is used to ensure sharp but smooth transitions at the boundaries of the coated region and the uncoated membrane.

**Figure 7.**
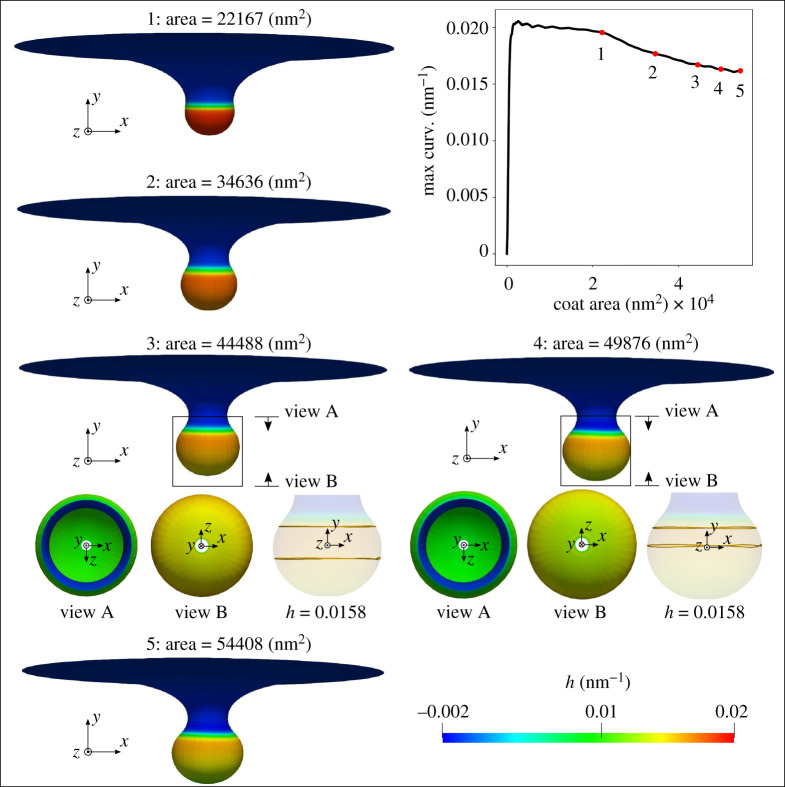
Formation of membrane buds with applied spontaneous curvature and increasing coat area. A surface tension $\gamma =0.002\;{\mathrm{pN}\;\mathrm{nm}}^{-1}$ is applied at the outer rim of the membrane. The coated region has a spontaneous curvature $h_{0}=0.02\;{\mathrm{nm}}^{-1}$, corresponding to a curvature radius of 50 nm. As illustrated by snapshots of the membrane at five different simulation stages, each with increasing area, the membrane progresses from a flat shape to a bud-like shape with increasing coated area. The evolution of the maximum curvature curve is plotted, which is smooth throughout the simulation. Symmetry breaking is observed in this simulation, as the curvature contour plots at stage 3 and 4 with $h=0.0158\;{\mathrm{nm}}^{-1}$ in the x--y plane are not straight in the y-direction. (Online version in colour.)

**Figure 8.**
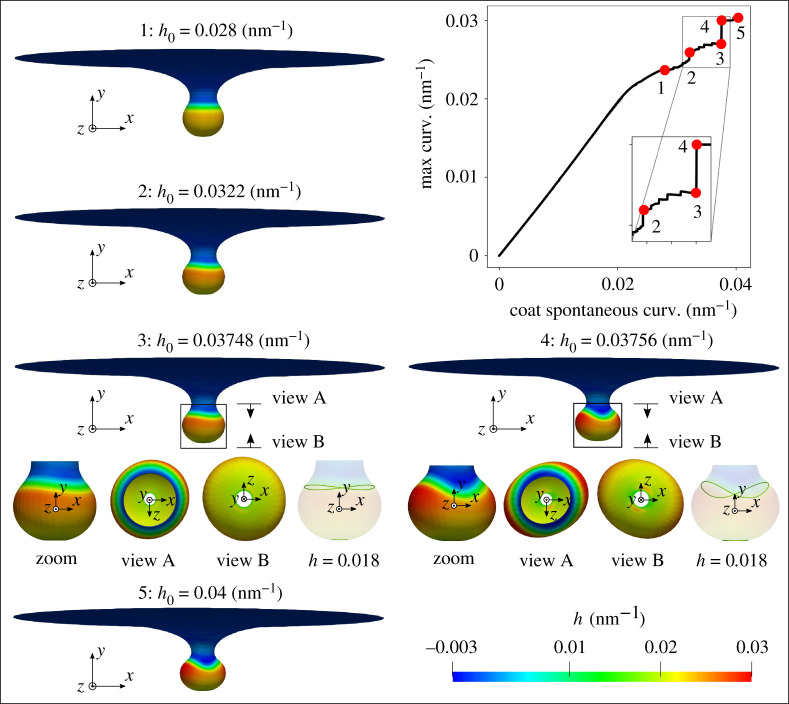
Formation of membrane buds with coat area and increasing spontaneous curvature. A surface tension $\gamma =0.002\;{\mathrm{pN}\;\mathrm{nm}}^{-1}$ is applied at the outer rim of the membrane. The coated region has a fixed radius of 80 nm. As illustrated by snapshots of the membrane at five different simulation stages, each with increasing $h_{0}$, the membrane progresses from a flat shape to a bud-like shape with increasing $h_{0}$. The curved curvature contour plot in the y-direction in the x--y plane at stage 3 with $h=0.018\;{\mathrm{nm}}^{-1}$ indicates the existence of symmetry breaking in the simulation. The sudden change of the maximum curvature curve between stage 3 and 4 indicates a growth of the associated instability in this simulation set-up, which is elaborated by the insets of simulation results at stage 3 and 4. (Online version in colour.)

The simulation results from both set-ups are reported in figures 7 and 8, where the coated membrane progresses from a flat shape to a bud-like shape. In addition, symmetry breaking of the membrane is observed in both cases. This is confirmed by the curved contour plot insets of h in the x--y plane in figures 7 and 8, which would otherwise be straight lines, indicating constant heights in the y-direction. Figures 7 and 8 further show that an additional instability mode exists in case (ii), where a rapid change in the maximum curvature curve between stage 3 and 4 occurs. It is worth mentioning that the spatial location of the maximum curvature evolves during the simulation. The different behaviour of the maximum curvature curve in figures 7 and 8 indicates that the two simulations possess different energetic paths for their solutions.

**Figure 9.**
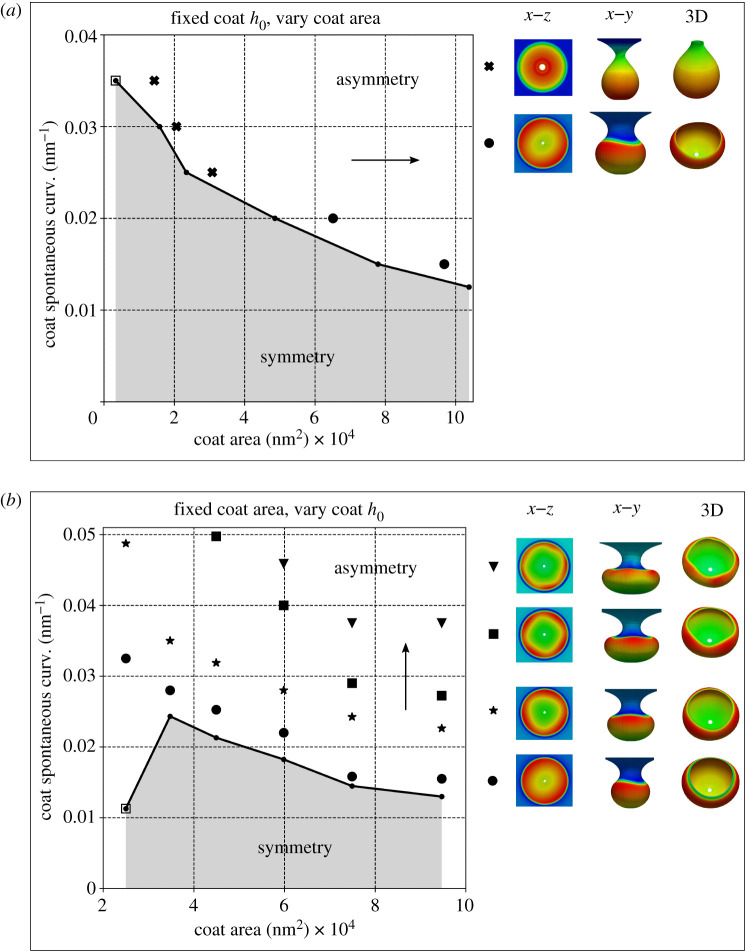
Membrane morphology evolution phase diagram for (*a*) similar simulation set-up as in figure 7 with fixed discrete $h_{0}$ but increasing protein-coated area, (*b*) similar simulation set-up as in figure 8 with fixed discrete protein-coated area but increasing $h_{0}$. The arrows indicate the progressively increasing quantities. The asymmetry morphology patterns differ for these two simulation set-ups. For case (*a*), both twisting (cross) and twofold (dot) wave shapes were captured in the asymmetry region. For case (*b*), twofold (dot), threefold (star), fourfold (square) and fivefold (triangle) shapes were captured in the asymmetry region. Representative bud shapes coloured by curvature values are shown in different views, where x--z view is in the undeformed configuration, and x--y view and 3D view are in the deformed configuration. The dots with empty surrounding square indicate the cases where the proposed symmetry-breaking criterion does not hold. (Online version in colour.)

Next, we conducted six simulations for each case to construct the membrane morphology evolution phase diagram. In each simulation of case (i), a different value of $h_{0}$ is assigned to the coated region. For a given value of $h_{0}$, we progressively increase the area of the coated region at an identical increment for all the simulations to allow the bud to form. In each simulation of case (ii), the radius of the coated region was set to a different value and then $h_{0}$ of the coated region was progressively increased at an identical increment for all the simulations to allow the bud to form. The membrane morphology evolution phase diagrams for both simulation set-ups appear in figure 9 with arrows indicating progressively increasing quantities, where different patterns appear in the asymmetric region. To detect the symmetry breaking in each simulation, first, we uniformly sample 20 discrete values of $u_{y}$ between its minimum and maximum at each increment of the coat area for case (i) or the coat $h_{0}$ for case (ii). Next, the range of the curvature h, $\left[h_{\text{min}},h_{\text{max}}\right]$, at every discrete $u_{y}$ is computed. For those heights with $h_{\text{min}}>0$, the relative change of h, denoted as $\Delta h=2(h_{\text{max}}-h_{\text{min}})/(\text{abs}(h_{\text{max}})+\text{abs}(h_{\text{min}}))$, is computed. At each incremental step, we thus have multiple values of Δh. Then the median value of Δh, denoted as $\Delta h_{\text{med}}$, is computed for that step. Now, for each simulation, we have an array of $\Delta h_{\text{med}}$, whose length is equal to the total number of incremental steps of either the coat area or the coat $h_{0}$. Our results show that symmetry breaking usually can be detected when $\Delta h_{\text{med}}$ is at its minimum over increments of coat area or $h_{0}$, pointing to a close to uniform value of Δh for that increment. We remark that this is a loose criterion for detecting symmetry breaking, as not all the symmetry-breaking events occur precisely at the loading step where $\Delta h_{\text{med}}$ is minimal. However, this criterion generally provides a consistent indication of symmetry breaking compared with our visual observation. Detailed discussion on the chosen symmetry-breaking criteria is provided in the electronic supplementary material.

The associated values of $h_{0}$ and the area of the coated region at the specific incremental step where $\Delta h_{\text{med}}$ is at its minimum are used to construct the phase diagram in figure 9. The dots with an empty surrounding square in figure 9 indicate the cases where the proposed symmetry breaking criterion does not hold. In the asymmetry region, not all the asymmetric patterns could be captured by the numerical simulations due to loss of numerical convergence during the solution iterations. This is due to the ill-conditioning of the system Jacobian matrix, that in turn is caused by the severe geometric and material nonlinearity, potentially including bifurcation points, in the vicinity of asymmetric deformation modes. We use standard unconstrained optimization methods like arc-length and trust-region to achieve convergence of the solution iterations, whenever possible, and report successfully captured asymmetric patterns. The placement of the reported patterns are shown in figure 9. Here, the same markers are chosen to denote similar shapes. The fact that our computational framework could capture the symmetry-breaking behaviour, and even the pattern changes from twofold to threefold/fourfold/fivefold, demonstrates the advantages of the proposed three-dimensional model over a reduced order axisymmetric model [19,20,81].

## 4. Discussion

Biomembranes play central roles in various cell-scale and organelle-scale phenomena like locomotion of cells [2], packaging and trafficking of nutrients and signalling constituents [4], maintaining organelle morphology and functionality [6–8], etc. In almost all these processes, these surfaces are known to undergo significant deformation through bending; and the evolution of the out-of-plane bending deformation is a key mechanism of morphological evolution, besides in-plane fluidity. Thus, many analytical and numerical approaches exist in the literature to model bending and curvature generation, especially for solving the governing equations resulting from the Helfrich-Canham [14] characterization of membrane elasticity.

While these widely used analytical and numerical approaches (e.g. Monge parametrization, arc-length parametrization and asymptotic methods) yield solutions to a wide range of boundary value problems of membrane bending, they are intrinsically limiting in capturing the complete envelope of membrane deformations due to the underlying axisymmetric restrictions on the kinematics and boundary conditions. Since the study of biomembrane deformation draws heavily from the well-established models of elastic shells [14,27], it is only natural to look for the validity of axisymmetric approximations and for the existence of non-axisymmetric solutions in the deformation of elastic shell geometries. Interestingly, many classical elastic structures have intrinsic unstable modes (eigen modes) that lead to a snap-through buckling like deformation or collapse of structures and are associated with lower deformation energy than the corresponding axisymmetric (non-buckling) modes of deformation. Such modes are ubiquitous in elastic shells and manifest as barrelling modes of thin cylinders [84], snap-through of elastic columns [85], and in folding, wrinkling and creasing of elastic membranes [86], etc. Notably, they have lower symmetry than the fully axisymmetric deformations. If such modes exist, and are accessible in biomembranes, then they would naturally lead to a reduction in the load and energy barriers to membrane deformation, and may result in heretobefore numerically unexplored deformation profiles and membrane morphologies. Accessing these lower symmetry modes and predicting the complex, three-dimensional deformation profiles in biomembranes provided the primary motivation for developing the computational framework presented in this work.

We note that the first application of this framework was in our recent study demonstrating how non-axisymmetric buckling lowers the energy barrier associated with membrane neck constriction in biomembranes [21]. In that study, we used a mechanical model of the lipid bilayer to systematically investigate the influence of location, symmetry constraints and helical forces on membrane neck constriction. Simulations from our model demonstrated that the energy barriers associated with constriction of a membrane neck are location-dependent and are significantly affected by kinematic constraints on the deformation. Importantly, if symmetry restrictions on the membrane deformation are relaxed, the constriction pressure and thus the energy barrier for constriction are dramatically lowered. Our studies established that despite different molecular mechanisms of neck formation in cells, the mechanics of constriction naturally leads to a loss of symmetry and occurs at a much lower load/energy threshold. Motivated by the improved understanding of membrane deformation and the undesired effects of axisymmetry restrictions observed in that study, we have further developed the framework and expanded its scope to modelling other important membrane deformation processes.

Accordingly, in this work, we model three classical biomembrane problems: formation of tubular shapes and their lateral constriction, Piezo1-induced membrane footprint generation, and budding of membranes during endocytosis. For each of these problems, we are able to validate against results and observation available in the literature for the simpler deformation modes, and also predict the more complex, less symmetric deformation profiles that are not accessible by the traditional analytical methods and axisymmetric numerical methods. Moreover, for the problem of endocytic vesicle budding, we also map a phase diagram classifying the symmetric and less-symmetric states.

The computational framework is implemented as an open-source software library and provided as a resource to the biophysics community. It is expected that this framework will serve as a platform for exploring complex deformation mechanisms (including geometric bifurcations and post-bifurcation responses) in biomembranes, and result in an improved understanding of the mechanics underlying various biomembrane phenomena. Future extensions envisioned are support for in-plane fluidity [60], surface diffusion (to model protein transport on the membrane), and a contact model (to model membrane–membrane interactions). In addition, the inability of the current framework to apply non-uniform Dirichlet boundary conditions and constraints on displacement degrees of freedom inside the domain (i.e. at non-interpolatory knots of the spline surface) are significant limitations and will be addressed in future developments.

## Data accessibility

This article has no additional data.

## Appendix A. Supporting information

### (a) S1. Text

**Mathematical framework and numerical formulation**

### (b) S2. Text

**Endocytosis boundary value problem details**
